# Cenp-E inhibitor GSK923295: Novel synthetic route and use as a tool to generate aneuploidy

**DOI:** 10.18632/oncotarget.4879

**Published:** 2015-08-06

**Authors:** Ailsa Bennett, Beatrice Bechi, Anthony Tighe, Sarah Thompson, David J. Procter, Stephen S. Taylor

**Affiliations:** ^1^ Faculty of Life Sciences, University of Manchester, United Kingdom; ^2^ School of Chemistry, University of Manchester, United Kingdom

**Keywords:** Chromosome Section, spindle assembly checkpoint, Mps1, aneuploidy, chromosome instability, Cenp-E

## Abstract

Aneuploidy is a common feature of cancer, with human solid tumour cells typically harbouring abnormal chromosome complements. The aneuploidy observed in cancer is often caused by a chromosome instability phenotype, resulting in genomic heterogeneity. However, the role aneuploidy and chromosome instability play in tumour evolution and chemotherapy response remains poorly understood. In some contexts, aneuploidy has oncogenic effects, whereas in others it is anti-proliferative and tumour-suppressive. Dissecting fully the role aneuploidy plays in tumourigenesis requires tools and facile assays that allow chromosome missegregation to be induced experimentally in cells that are otherwise diploid and chromosomally stable. Here, we describe a chemical biology approach that induces low-level aneuploidy across a large population of cells. Specifically, cells are first exposed to GSK923295, an inhibitor targeting the mitotic kinesin Cenp-E; while the majority of chromosomes align at the cell's equator, a small number cluster near the spindle poles. By then driving these cells into anaphase using AZ3146, an inhibitor targeting the spindle checkpoint kinase Mps1, the polar chromosomes are missegregated. This results in, on average, two chromosome missegregation events per division, and avoids trapping chromosomes in the spindle midzone, which could otherwise lead to DNA damage. We also describe an efficient route for the synthesis of GSK923295 that employs a novel enzymatic resolution. Together, the approaches described here open up new opportunities for studying cellular responses to aneuploidy.

## INTRODUCTION

Aneuploidy is defined as a karyotype with a chromosome number that deviates from the expected. For example, individuals with Down Syndrome have three copies of chromosome 21 instead of two, leading to developmental disabilities and shortened life expectancy. Aneuploidy can arise due to unequal chromosome segregation during mitosis and meiosis, generating daughter cells with extensive gene copy number changes. The consequences of the chromosomal imbalance manifest at the cellular level. In yeast, aneuploidy induces proteotoxic stress, suppresses proliferation and reduces fitness [1–4]. Similarly, in mammalian cells, aneuploidy is anti-proliferative and sensitizes cells to compounds that interfere with protein folding [5–7].

Despite aneuploidy's anti-proliferative potential, it is a common feature of cancers and indeed, Boveri suggested over 100 years ago that aneuploidy might promote tumour formation [8–10]. In mice, artificial induction of aneuploidy can act either as a tumour promoter or a tumour suppressor, depending on the context [11–13]. An emerging view is that while a low level of aneuploidy provides enough genetic variation to fuel tumour evolution, excessive chromosome instability creates genetic chaos, which is detrimental to fitness [14, 15]. Nevertheless, because aneuploidy is anti-proliferative in non-transformed cells, understanding how cancer cells tolerate aneuploidy is a key question.

Defining the acute and chronic effects of chromo- some missegregation requires tools and assays to generate aneuploidy in otherwise diploid, chromosomally stable cells so that the short and long term consequences on cellular physiology can be studied. A current approach to induce chromosome missegregation involves arresting cells in mitosis with drugs that block spindle assembly, e.g. the microtubule targeting agent nocodazole or Eg5/KSP kinesin inhibitors such as monastrol [16–19]. Following washout, spindle assembly leads to chromosome segregation but with maloriented chromosomes that missegregate [16]. While effective, a monastrol-washout has a major impact on spindle assembly and only gives rise to one chromosome missegregation event every three divisions [19]. Moreover, these chromosomes can often get trapped in the cleavage furrow or form micronuclei, leading to DNA damage, in turn causing chromosome translocations as well as whole chromosome aneuploidies [20, 21]. Other methods of generating aneuploidies includes the use of topoisomerase II inhibitors [22], but again this approach induces DNA damage leading to chromosome translocations. Dissecting aneuploidy without the complication of DNA damage therefore requires new approaches.

Cenp-E (Centromere Associated Protein-E), is a plus-end directed kinesin-7 motor protein, required for chromosome segregation in both mitosis and meiosis [23]. Cenp-E localises to kinetochores throughout mitosis, with phosphorylation by Aurora kinases A and B, plus the opposing function of protein phosphatase 1, imposing important regulatory control [24–26]. Cenp-E function aids chromosome alignment by moving chromosomes from the spindle poles to the metaphase plate [25]. Specifically, by linking the unattached kinetochores on mono-oriented chromosomes to an adjacent, mature kinetochore fibre, Cenp-E mediates congression of polar chromosomes prior to biorientation [27].

When Cenp-E expression is perturbed using antibody injections, immunodepletions, anti-sense, siRNA or gene deletion approaches, complete chromosome alignment is inhibited [28–30]. This is consistent with even a single unattached kinetochore being sufficient to prevent anaphase onset [31–33]. Cenp-E inhibition leads to persistent activation of the spindle assembly checkpoint (SAC), in turn leading to a mitotic arrest [29, 34, 35]. Cenp-E may also play a direct role in the SAC; Cenp-E binds and, in the absence of bound microtubules, activates the SAC kinase BubR1 [36, 37].

In the quest to develop novel antimitotic chemotherapy agents, mitotic kinesins are attractive targets [38]. To explore Cenp-E's potential, small molecule inhibitors that disrupt Cenp-E function have been developed [39, 40]. A high throughput library screen seeking compounds that inhibited the microtubule-stimulated ATPase activity of Cenp-E led to the development of GSK923295 [40]. GSK923295 is an allosteric inhibitor of Cenp-E that prevents ATP hydrolysis, thus stabilizing the enzyme in a conformation with increased affinity for microtubule binding [41]. Cells treated with GSK923295 assemble bipolar spindles and align most of their chromosomes. However, a number remain clustered near the spindle poles, leading to SAC arrest and apoptosis [41].

We reasoned that in combination with drugs that override the SAC, GSK923295 would be a useful tool to efficiently generate whole chromosomes aneuploidies, without the risk of DNA damage. Here we describe a novel approach to synthesize GSK923295, together with an assay that induces on average two chromosome missegregation events per cell division without trapping chromosomes in the cleavage furrow.

## RESULTS

### Enzymatic resolution facilitates an asymmetric synthesis of GSK923295

**Figure 1:**
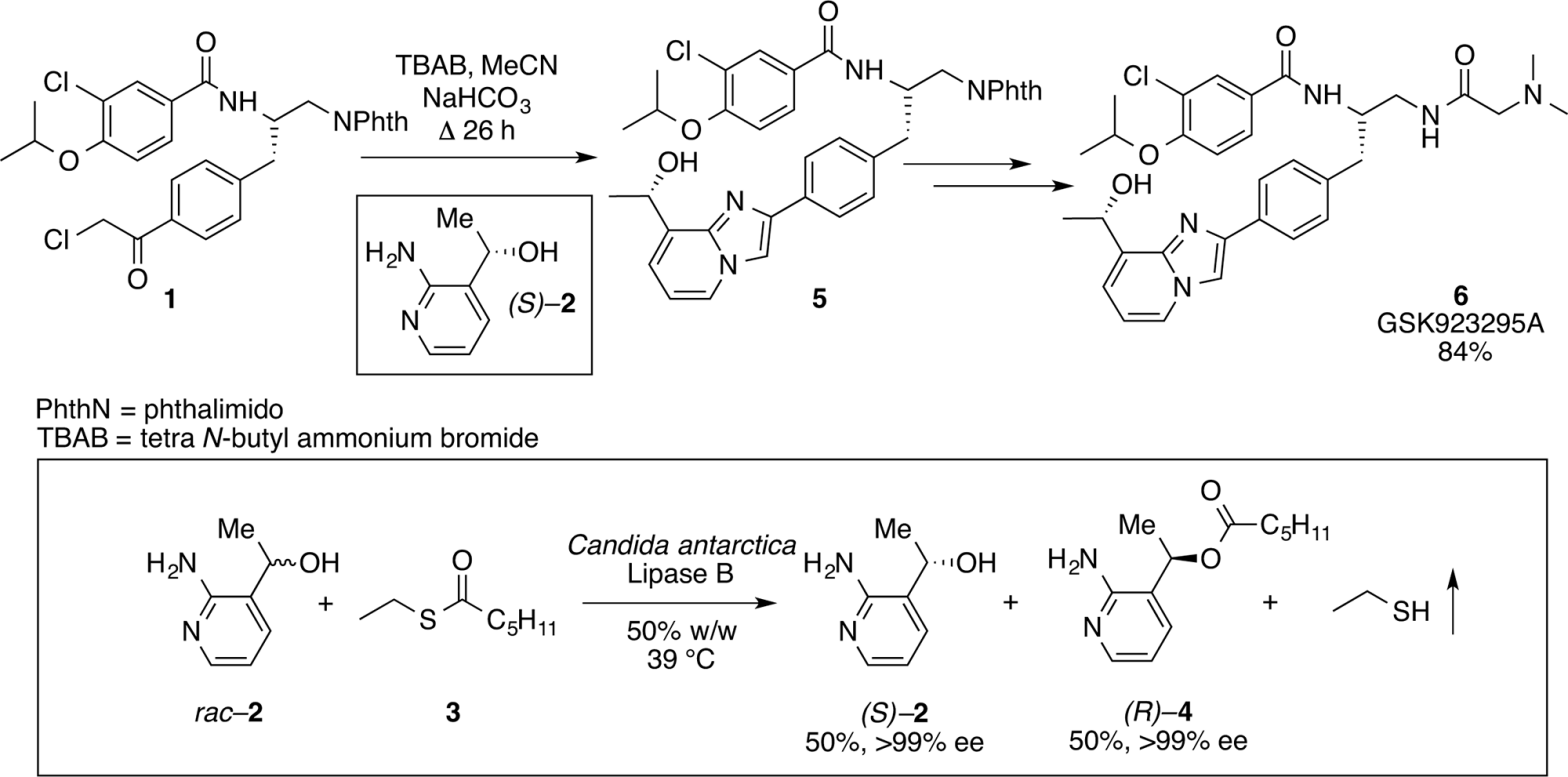
Resolution of racemic mixture to synthesise GSK923295 The desired (*S*)-enantiomer (**2**) was obtained by the resolution of the racemic mixture of 1-(2-amino-3-pyridinyl)ethanol (*rac*-**2**), through the reaction with S-ethyl thiohexanoate at 39°C with *Candida Antarctica* Lipase B enzyme (50% w/w). The pure enantiomer (**2**) and the intermediate (**1**) then reacted to give the pyridyl imidazole (**5**), with subsequent reactions yielding GSK923295 (**6**).

To experimentally induce whole chromosome aneuploidies, we set out to synthesise the Cenp-E inhibitor, GSK923295 by following previously published routes [40, 42]. A key step involves resolution of racemic 1-(2-amino-3-pyridinyl)ethanol (Fig. 1, compound **2**) to obtain enantiomerically pure 1*S*-(2-amino-3-pyridinyl)ethanol (*(S)–***2**) for subsequent reaction with an intermediate (**1**), ultimately producing GSK923295 (**6**). In our hands, the published preparative HPLC resolution method was inefficient and therefore we explored other options. Previously, a mutated variant of *Candida antarctica* lipase B was shown to successfully resolve aromatic secondary alcohols [43], so we explored a similar strategy. *Candida antarctica* lipase B (CALB) enzyme was added to a racemic mixture of (**2**), with excess S-ethyl thiohexanoate **3**, under solvent free conditions, at 39°C (Fig. 1). The progress of the reaction was monitored using HPLC. The enzyme specifically reacted with the (*R*)-enantiomer over a 12-hour period, leaving the (*S*)-enantiomer unreacted. The ester and alcohol were each obtained in 50% yield, and the alcohol *(S)*–**2** in > 99% enantiomeric excess (ee). Purification by column chromatography then yielded pure 1*S*-(2-amino-3-pyridinyl)ethanol (*(S)*–**2**). The pure (*S*)-enantiomer was then reacted with phenacyl chloride (**1**) to give the pyridyl imidazole (**5**). Methods used for subsequent steps to GSK923295 (**6**) were as published [44–47]. Our synthetic studies employing a lipase in a kinetic resolution, illustrate the (*R*) specificity of the enzyme and provide a convenient and reliable synthesis of the Cenp-E inhibitor GSK923295.

### GSK923295 inhibits chromosome alignment

**Figure 2:**
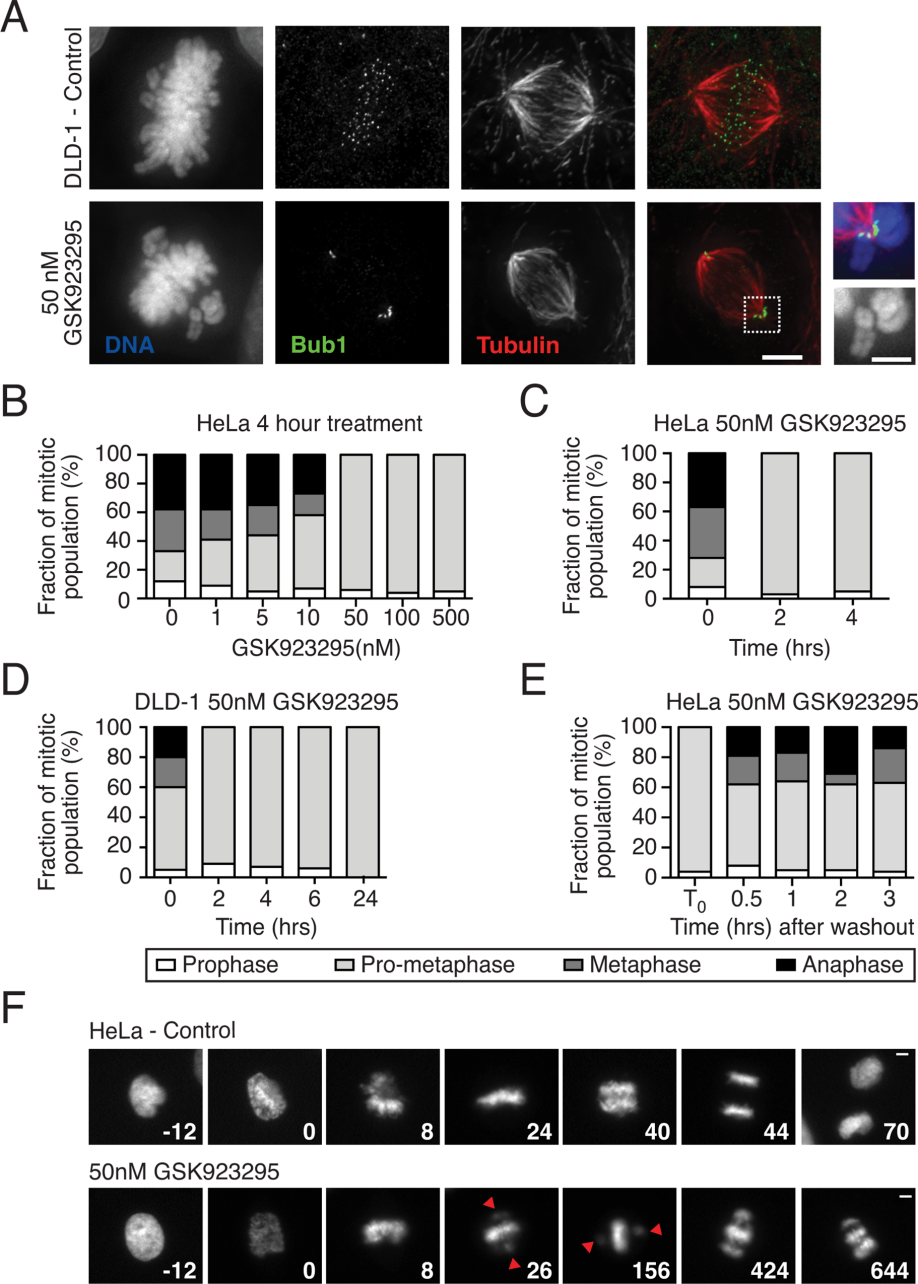
GSK923295 induces chromosome misalignment **A.** Immunofluorescence images of DLD-1 cells treated with 50 nM GSK923295 for four hours, fixed and stained for DNA (blue), Bub1 (green) and tubulin (red), with insets showing unaligned chromosome(s). Bar: 5 μm or 2.5 μm for increased magnification. **B.** A bar chart quantifying the number of cells in each mitotic phase following exposure of HeLa cells to GSK923295 at various concentrations. **C.** Quantification of the number of HeLa cells in each mitotic phase over a time course, following 50 nM GSK923295 treatment. **D.** The number of DLD-1 cells in each stage of mitosis over time after 50 nM GSK923295 treatment. **E.** Treatment of HeLa cells with 50 nM GSK923295, and classification of 100 mitotic cells after no washout (control), or at various times following wash-out. **F.** Time lapse sequences of control and 50 nM Cenp-E inhibitor treated HeLa GFP Histone-H2B cells. Time zero represents nuclear envelope break down. Bar: 5 μm.

To characterise the synthesized inhibitor, diploid DLD-1 colon cancer cells were treated with 50 nM GSK923295. After four hours, cells were fixed and stained to detect Bub1, tubulin and the DNA, then analysed by immunofluorescence microscopy (Fig. 2A). Consistent with previous reports [41], in the presence of GSK923295, bipolar spindles formed and while the majority of chromosomes aligned at metaphase, a few remained close to the spindle poles. The kinetochores of these unaligned chromosomes stained strongly for Bub1, indicating that they were not correctly attached to spindle microtubules [48, 49]. Thus, our preparation of GSK923295 yields the expected cellular phenotype.

To identify minimal concentrations and exposure times required to induce a potent chromosome misalignment phenotype, HeLa cells were first treated with varying concentrations of GSK923295 for four hours then fixed and analysed by fluorescence microscopy (Fig. 2B). 100 mitotic cells for each condition were assigned to one of four different stages of mitosis, namely prophase, prometaphase, metaphase and anaphase. Cells with unaligned chromosomes were classified as prometaphase. In the control populations, on average 12% were in prophase, 21% prometaphase, 29% metaphase and 38% in anaphase. At 50 nM, the number in prometaphase increased to 94%, with no obvious metaphase or anaphase figures (Fig. 2B). Many of these prometaphase figures typically had bipolar spindles with many aligned chromosomes but a few polar chromosomes, similar to the situation in DLD-1 cells (Fig. 2A). This phenotype did not appear to change at concentrations above 50 nM. Next, we treated HeLa cells with 50 nM GSK923295 then analysed them at various time points as described above. Within two hours of treatment, > 95% of cells were in prometaphase, indicating a complete block of metaphase and anaphase (Fig. 2C). We observed a similar result in DLD-1 cells, with ∼91% of cells scored as prometaphase by two hours of exposure (Fig. 2C). Thus, in both HeLa and DLD-1 cells, a two-hour exposure of 50 nM GSK923295 is sufficient to induce a potent chromosome misalignment phenotype.

We next set out to determine whether GSK923295-mediated inhibition of Cenp-E is reversible by asking whether cells completed chromosome alignment following drug washout. HeLa cells were treated with 50 nM GSK923295 for four hours, washed twice with PBS then fresh media added. At various time points, chromosome alignment was analysed as above (Fig. 2D). In the control population, where the inhibitor was not washed out, 96% of the mitotic cells were scored as prometaphase. By contrast, following a 30-minute period after washout, 54% were classified as prometaphase, 19% metaphase, and 19% anaphase. Therefore, following washout of GSK923295 chromosome alignment appears to recover. Moreover, the presence of anaphase figures indicates SAC satisfaction. Thus, Cenp-E function can be restored following washout of GSK923295, indicating that the drug is reversible.

### GSK923295 induces mitotic arrest

The lack of obvious metaphases and anaphases in GSK923295-treated cultures is consistent with persistent activation of the SAC and mitotic arrest. Indeed, Cenp-E inhibition has been shown to activate the SAC [23, 29]. However, it is conceivable that GSK923295-treated cells also slip out mitosis without completing chromosome alignment and undergoing sister chromatid disjunction [50]. To distinguish between these two possibilities, we turned to time-lapse microscopy, analysing HeLa cells expressing a GFP-tagged histone to visualize the chromosomes in living cells (Fig. 2F). HeLa GFP Histone-H2B cells were treated with 50 nM GSK923295 then analysed by time-lapse fluorescence microscopy, acquiring images every 2 minutes, marking nuclear envelope break-down (NEBD) at time zero. In the representative control cell shown in Fig. 2F, chromosome alignment was complete by 24 minutes and chromosome segregation apparent by 40 minutes, such that by 70 minutes the daughter cells had returned to interphase. In the GSK923295-treated cell, most chromosomes had aligned by 26 minutes but at least 1 chromosome was visible near each of the two spindle poles. This configuration persisted for several hours until chromosomes started to “fall off” the metaphase plate, resulting in more chromosomes/chromatids near the spindle poles (Fig. 2F, see 424 and 644 mins). This is highly reminiscent of cohesion fatigue [51, 52], a phenomenon whereby aligned chromosomes are eventually peeled apart by spindle forces, during a metaphase delay. Note that the HeLa cell line used here is particularly prone to cohesion fatigue [51–53]. Once cohesion fatigue occurs, satisfaction of the SAC is impossible and indeed, the cell shown eventually underwent slippage (not shown), but other cells in the population were shown to undergo apoptosis. Thus, GSK923295-mediated inhibition of Cenp-E does indeed lead to a prolonged mitotic arrest, which in the first instance appears to be caused by blocking complete chromosome congression.

### GSK923295 induces death in mitosis and post-mitotic apoptosis

**Figure 3:**
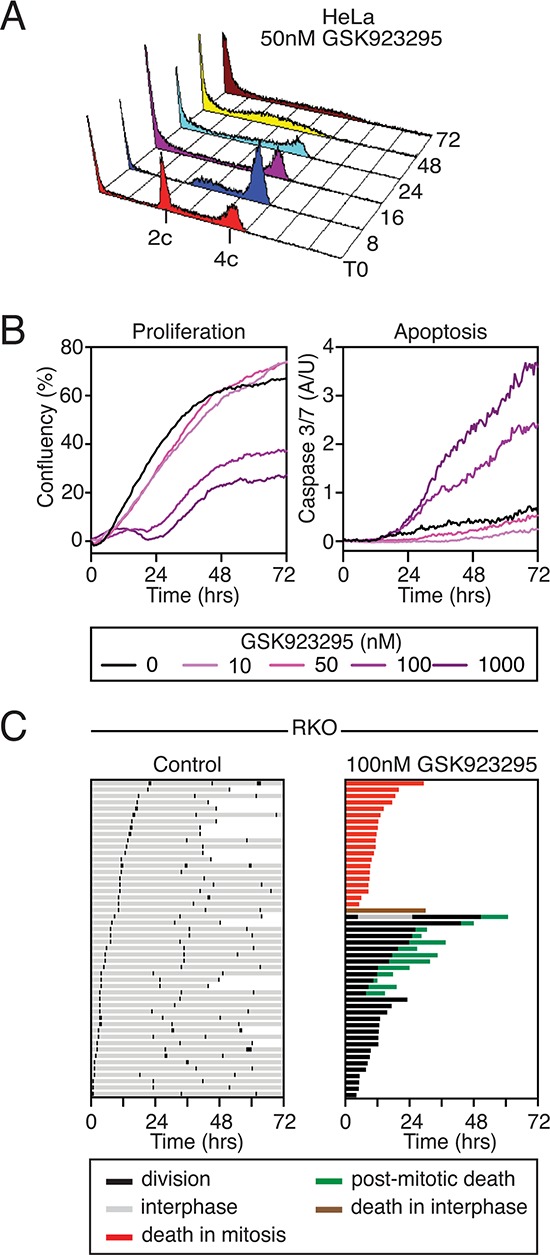
GSK923295 causes cell death **A.** DNA content histograms of HeLa cells treated with 50 nM GSK923295, over a time course. **B.** Line graph showing percent confluency and caspase 3/7 activation of RKO cells treated with varying Cenp-E inhibitor concentrations. **C.** Fate profiles for untreated and Cenp-E inhibitor treated RKO cells. One bar represents one cell.

Cell fate in response to anti-mitotic agents varies considerably depending on the cell line studied, the anti-mitotic drug used and the drug concentration applied [54]. Moreover, genetically identical cells can undergo different fates despite identical environmental conditions. The time-lapse analysis above shows that HeLa cells undergo a prolonged mitotic arrest, then cohesion fatigue followed by mitotic exit (in the representative example). To determine whether this was the dominant phenotype, HeLa cells treated with 50 nM GSK923295 were analysed by flow cytometry at various time points to determine DNA content (Fig. 3A). After an eight-hour exposure, the vast majority of cells had 4c DNA contents, consistent with an inability to undergo a normal cell division. At later time points, and in particular by 48 hours, the majority of cells had sub-2c DNA contents, indicating extensive apoptosis (Fig. 3A). Thus, although 50 nM GSK923295 only initially leads to misalignment of a few chromosomes (Fig. 2A), which are in principle capable of alignment (Fig. 2E), we suggest that the subsequent cohesion fatigue generates single chromatids that cannot align (Fig. 2F), thereby leading to persistent activation of the SAC, in turn leading to extensive apoptosis.

As mentioned above, the HeLa cells used here are particularly sensitive to cohesion fatigue [51, 52]. Thus, the prolonged mitotic arrest and subsequent death observed in may be a reflection of cohesion fatigue rather than prolonged Cenp-E inhibition. Therefore we turned to RKO cells, a diploid colon cancer cell line that appears to be more resistant to cohesion fatigue (not shown). When exposed to taxol, nocoazole or an Eg5 inhibitor, RKO cells typically undergo death in mitosis [54]. To determine the long-term effects of GSK923295 on RKO cells, time-lapse imaging was performed over a 72 hour period. Proliferation and apoptosis were analysed by confluence based measurements and caspase 3/7 fluorescent probes. While concentrations of 50 nM and below appeared relatively benign, 100 nM GSK923295 inhibited proliferation and induced apoptosis (Fig. 3B). To determine when cells treated with 100 nM were dying, we inspected the image sequences and used phase contrast morphology to monitor mitosis and generated cell fate profiles as described previously [54]. Briefly, each fate profile represents 50 cells with the colour of the line showing the fate of the cell in the period of imaging, and the length of the line is the time taken to undergo the particular cell behaviour. In the control population, cells underwent, on average, three cell divisions during 72 hours. Consistent with the HeLa time-lapse and FACS data, GSK923295 treated RKO cells underwent mitotic arrest. 40% of the cells then died in mitosis after an average arrest time of 11.91 hours. Cohesion fatigue was not obvious in these cells, but higher resolution time-lapse microscopy would be required to definitively conclude this. Strikingly, despite the continued presence of GSK923295, 26% of the cells divided after an arrest of 15.22 hours, yielding two daughter cells. Interestingly, only one of these cells entered a second mitosis. Of the rest, 24% died in the subsequent interphase, while the remainder remained arrested in interphase for the remainder of the experiment.

### Sequential Cenp-E and Mps1 inhibition generates aneuploid daughter cells

**Figure 4:**
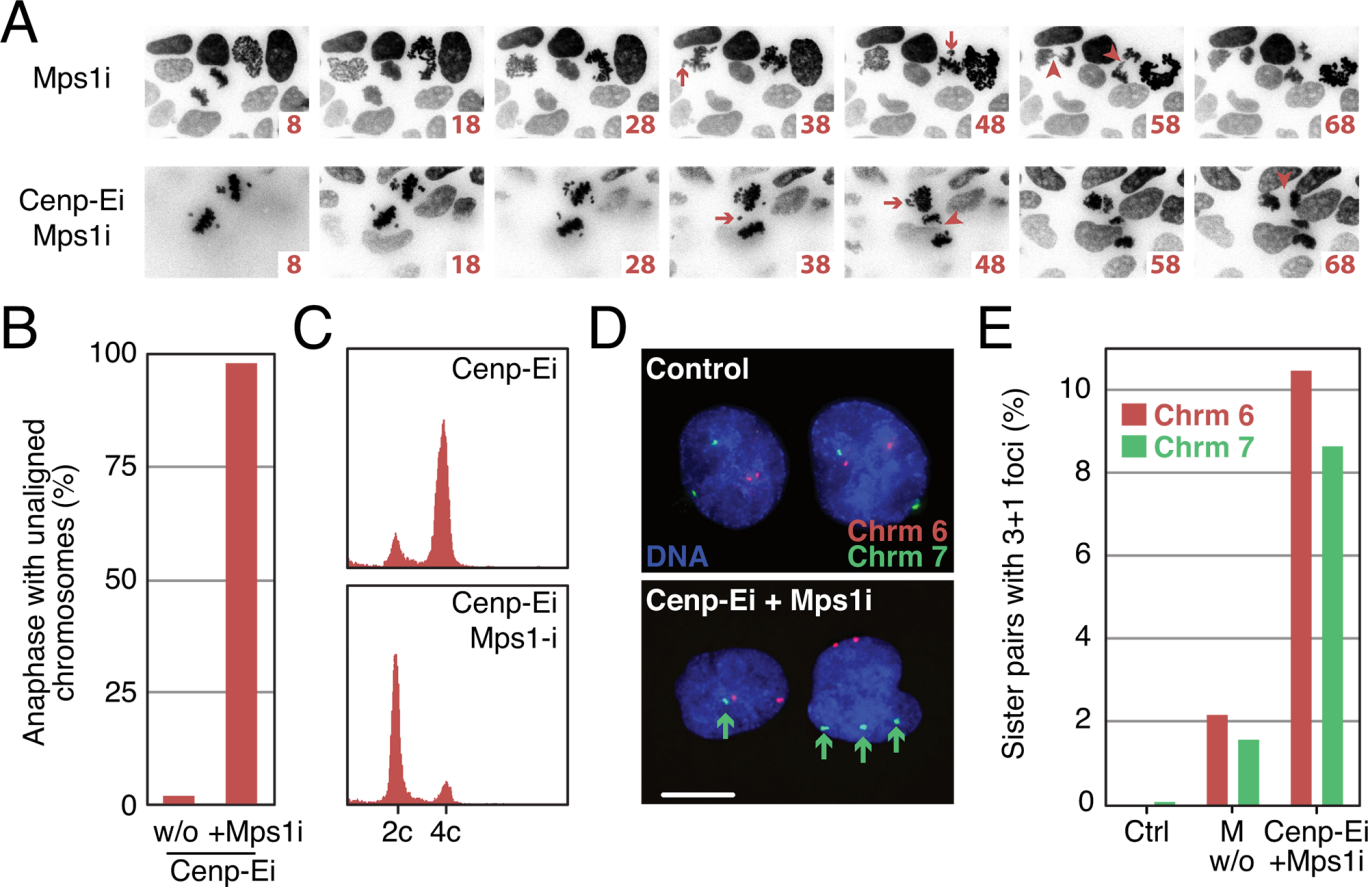
Sequential Cenp-E and Mps1 inhibition generates aneuploid daughter cells **A.** Time lapse sequences of DLD-1 histone-H2B-mCherry cells treated with the Mps1 inhibitor alone or Cenp-E inhibitor treated cells, washed into the Mps1 inhibitor. Arrows indicate mitotic defects: unaligned chromosomes/lagging chromosomes. **B.** Quantification of the number of anaphases in HCT-116 cells with unaligned chromosomes after treatment with Cenp-E inhibitor alone or Cenp-E inhibitor and Mps1 inhibitor combined. **C.** DNA content histograms of Cenp-E and Cenp-E and Mps1 inhibitors treated cells. **D.** FISH analysis labelling chromosomes 6 and 7 in control and Cenp-E and Mps1 inhibitor treated cells. Bar: 10 μm. **E.** Bar chart quantifying the number of pairs of daughter cells with 3+1 foci under control, monastrol washout (100 μM), and Cenp-E and Mps1 inhibitor treated conditions.

Our analysis confirms that GSK923295-treated cells assembly bipolar spindles and, while the majority of chromosomes align, a few remain clustered near the spindle poles. We reasoned that driving these cells into anaphase by overriding the SAC should induce missegregation of the polar chromosomes, thereby generating aneuploid daughter cells. To test this, DLD-1 Histone-H2B- mCherry cells were treated with 50 nM GSK923295 for 4 hours and then analysed by time-lapse imaging (Fig. 4A). Metaphase cells with polar chromosomes were identified and 2 μM of AZ3146, an Mps1 kinase inhibitor, was added to override the SAC [55]. As predicted, AZ3146 induced anaphase onset in the cells with polar chromosomes leading to obvious non-disjunction events (Arrows in Fig. 4A, Supplemental Movie S1). Importantly, lagging chromosomes were not observed (Arrowheads in Fig. 4A, lower panels). By contrast, addition of AZ3146 in the absence of GSK923295 resulted in anaphases with chromosomes near the poles and in the midzone that appeared to get stretched between the two separating masses (Fig. 4A, Supplemental Movie S2). To confirm that this strategy also induces chromosome missegregation in diploid cells we repeated the analysis in HCT116 cells. Quantitation of time-lapse sequences showed that 98% of the anaphases observed in GSK923295-AZ3146-treated cultures underwent anaphase with unaligned chromosomes (Fig. 4B). To confirm that these cells completed cytokinesis, HCT116 cells were exposed to GSK923295 for 4 hours and mitotic-arrested cells isolated by selective detachment. Flow cytometry analysis showed that the vast majority of the isolated cells had 4c DNA contents consistent with mitotic arrest (Fig. 4C). Two hours after addition of AZ3146 the vast majority of cells had 2c DNA contents, indicating that they had completed chromosome segregation and cytokinesis. To confirm that these cells were aneuploid, we performed *in situ* fluorescent hybridization on interphase cells following sequential GSK923295-AZ3146 exposure, using probes to detect the centromeres of chromosomes 6 and 7 (Fig. 4D). We focussed on cell pairs to enrich for daughters. In control populations, we typically saw two foci for each of the probes. In drug-treated populations, we often saw cell pairs where one cell had three foci for one of the probes while the adjacent cell only had 1, i.e. a 3+1 foci pattern (Fig. 4D), indicating a missegregation event. Quantitation showed that ∼9% of cells missegregated chromosome 6 or 7. By contrast, following a monastrol washout, only ∼2% of cells had 3+1 foci. Multiplying these rates by the total number of chromosomes per cell to calculate how often any chromosome missegregates indicates cells treated with sequential GSK923295-AZ3146 exposure missegregate ∼2 chromosomes per division, compared to monastrol washout where less than 50% of divisions result in a single missegregation event (42%). Missegregation in untreated populations is rare, with only one missegregation event in 100 divisions.

## DISCUSSION

Chiral amino alcohols are important building blocks for medicinal chemistry and drug discovery [56–58]. In particular, 1*S*-(2-amino-3-pyridinyl)ethanol *(S)–***2** is a crucial component in the synthetic route to GSK923295. However, access to amino alcohol motifs in enantiopure form, using either asymmetric synthetic methods or resolution, is often difficult thus reducing the overall efficiency of syntheses. We show here that an enzyme-based kinetic resolution using CALB, efficiently and reproducibly gives the maximum 50% yield of the required 1*S*-(2-amino-3-pyridinyl)ethanol (*S)***–2** in high enantiomeric excess. A previously reported route to GSK923295 involved resolution of *rac*–**2** by chiral HPLC [40]; a process that we found to be unsatisfactory. In turn, our asymmetric biocatalytic approach has resulted in an efficient and convenient route to GSK923295. Our novel asymmetric approach has the potential to deliver novel compounds inspired by GSK923295.

We set out to use GSK923295 in conjunction with the Mps1 inhibitor AZ3146 to efficiently induce whole chromosome aneuploidies without major disruption to the spindle and without inducing DNA damage. Consistent with previous reports [41], our data shows that GSK923295 does not prevent spindle assembly but efficiently and rapidly prevents complete chromosome alignment. Importantly, GSK923295 is reversible; following washout, the remaining chromosomes align and anaphase occurs in a timely manner. While this suggests that GSK923295 does not induce irreparable damage, note that prolonged mitotic arrest in the presence of a functional spindle can induce cohesion fatigue, which is irreversible [51, 52]. This can be avoided either by short mitotic arrests or selecting cell lines that are more resistant to cohesion fatigue. Indeed, when overriding the SAC in GSK923295-treated DLD-1 cells, the majority of chromosomes appeared to segregate normally. Importantly however, the polar chromosomes missegregated giving rise to aneuploid daughters. Although Cenp-E is required to maintain the integrity of kinetochore-microtubule interactions on bioriented chromosomes [59, 60], when we triggered anaphase in GSK923295-arrested cells, we did not observe lagging chromosomes or bridges. Thus, in contrast to nocodazole and monastrol washouts, the approach we describe is promising in terms of inducing whole chromosome aneuploidies without concomitant DNA damage. Moreover, interphase FISH indicates that the sequential GSK923295-AZ3146 exposure gave rise to ∼2 chromosome missegregation events per division, and is thus about five-fold more efficient than a monastrol-washout-based strategy.

The chemical biology approach we describe here to induce aneuploidy has several advantages over molecular genetic approaches. Small molecular inhibitors can efficiently induce highly penetrant effects across whole cell populations, facilitating, for example, large-scale bio- chemical experiments that are more difficult to achieve with RNAi. And indeed, we show that > 95% of cells treated with GSK923295-AZ3146 missegregate at least one chromosome. Inhibitors can easily be combined with other modalities, such as RNAi. This may facilitate RNAi screens for cellular responses to aneuploidy. Inhibitors inactivate their targets with rapid onset, allowing loss-of-function experiments to be conducted with precise temporal control. Consequently, the assay we describe here involves short time courses that can be used to avoid issues such as cohesion fatigue. Finally, small-molecule regimens can easily be transferred from one cell type or even species to another, including specialized cell types, which are not amenable to RNAi-based strategies. This could facilitate aneuploidy induction in specialised cell types, such as stem cells, or in existing lines expressing specific biosensors and reporters.

Interestingly, when RKO cells were exposed to GSK923295, 26% arrested in mitosis but then eventually divided. Whether these dividing cells aligned all their chromosomes before committing to anaphase, or whether they underwent anaphase with unaligned chromosomes is not known. Distinguishing between these two possibilities is important; the first possibility implies SAC satisfaction while the latter implies SAC exhaustion. High-resolution time-lapse imaging will be required to address this. Following division in the presence of GSK923295, the vast majority of cells then failed to undergo another mitosis, indicating a robust cell cycle arrest. Again, determining whether the dividing cells underwent anaphase with unaligned chromosomes or not will be required to determine why these cells engaged post-mitotic anti-proliferative responses. If chromosome segregation was completed, then it could be the prolonged arrest was sufficient to induce the subsequent G1 arrest [61]. If anaphase was initiated with unaligned chromosomes, then DNA damage and/or aneuploidy could be the cause [19, 20]. Distinguishing between these possibilities is an important area for future experimentation. Moreover, the chemical biology approaches we describe here have potential in terms of understanding how cells respond to chromosome missegregation and tolerate aneuploidy.

## MATERIALS AND METHODS

### Synthesis

All synthetic methods were as described previously [42]. All chemicals were purchased from Sigma. In a closed reaction vessel equipped with bleach trap, *S*-ethyl thiohexanoate (10 mmol) (**3**) was added to racemic 1-(2-aminophenyl)ethanol (1 mmol) (*rac*–**2**) and the CALB enzyme preparation (50% w/w). The reaction proceeded at 39°C and was monitored *via* HPLC analysis (Chiralcel OD-H; Heptane: ethanol 90:10, 0.1% IPAM; Flow: 0.8 mL/min). After completion at 12 hours, the enzyme was removed by filtration. The mixture was then purified by column chromatography (ethyl acetate as eluent) to give ester (*R)–***4** and the unreacted alcohol (*S)–***2**.


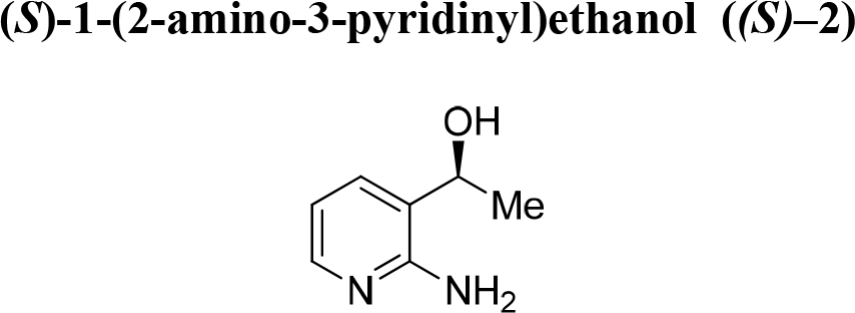


Unreacted alcohol (*S)–***2**. Colourless oil. RT = 10.8 min. Yield: 50%. *ee*: > 99%. [α]_D_ = −0.5° (ethanol, c = 1.00).


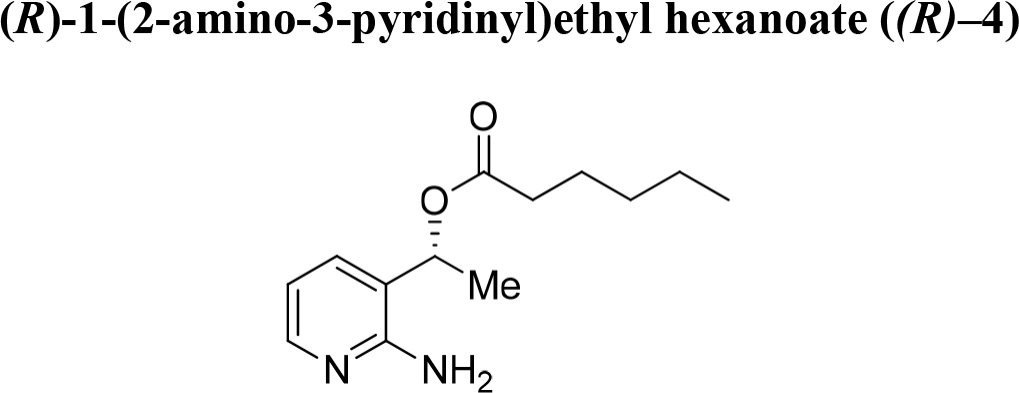


Ester (*R)–***4** obtained from reacting alcohol *(R)*–**2**. Colourless oil. RT = 7.1 min. Yield: 50%. ^1^H NMR (400 MHz, CDCl_3_) δ 0.78–0.86 (m, 3H, CO(CH_2_)_4_*CH_3_*), 1.18–1.30 (m, 4H, CO(CH_2_)_2_*(CH_2_*)*_2_*CH_3_), 1.49–1.59 (m, 5H, COCH_2_*CH_2_*(CH_2_)_2_CH_3_), OCH*CH_3_*), 2.23–2.29 (m, 2H, CO*CH_2_*(CH_2_)_3_CH_3_), 5.15 (s, 2H, *NH_2_*), 5.82 (q*, J* = 6.8 Hz, 1H, O*CH*CH_3_), 6.58 (dd, J = 5.2, 7.8 Hz, 1H, H-5), 7.40–7.41 (m, 1H, H-4), 7.86–7.88 (m, 1H, H-6). ^13^C NMR (100 MHz, CDCl_3_) δ 13.9 (CO(CH_2_)_4_*C*H_3_), 19.1 (*CH_3_*CHOH), 22.3 (CO(CH_2_)_3_*C*H_2_CH_3_), 24.6 (CO(CH_2_)_2_*C*H_2_), 31.1 (COCH_2_*CH_2_*CH_2_), 34.3 (CO*C*H_2_CH_2_), 68.9 (CH_3_*C*HOH), 113.6 (*C*H-5), 119.9 (*C*-3), 136.2 (*C*H-4), 146.4 (*C*H-6), 156.4 (*C*-2). 173.3 (*C*O).

### Cell lines

DLD-1, HeLa, RKO and HCT-116 cell lines were cultured in DMEM plus 10% fetal calf serum (LifeTechnologies), 2 mM glutamine, 100 U/mL penicillin, and 100 U/mL streptomycin (Lonza) at 37°C in a humidified 5% CO_2_ atmosphere. DLD-1 Histone-H2B-mCherry were as described previously [62]. Small molecule inhibitors dissolved in DMSO were as follows: GSK923295, Cenp-E inhibitor (in house); AZ3146, Mps1 inhibitor (Tocris); Monastrol, Eg5 inhibitor (Sigma).

### Immunofluorescence

Cells were plated at 8 × 10^4^ cells/mL on 19 mm (VWR International) coverslips at 500 μL. After overnight incubation, the Cenp-E inhibitor was added for various time periods. Cells were fixed with 1% formaldehyde, quenched with glycine, and then permeabilised with PBST (PBS and 0.1% Triton X-100). For microtubule staining the PEM buffer was used. Cells were pre-extracted with 100 mM Pipes, 1 mM MgCl_2_, 0.1 mM CaCl_2_, and 0.1% Triton X-100 for 90 seconds, followed by fixation with 4% formaldehyde in PEM buffer for 10 minutes. Cells were then incubated with sheep anti-Bub1 SB1.3 [49] and with mouse anti-tubulin TAT1 [63], for 30 minutes, and then washed and incubated with secondary antibodies Cy2-, and Cy3-, antisheep/mouse (Millipore) for 30 minutes. Hoechst 33358 (Sigma) at 1 μg/mL was then added to the cells, followed by mounting onto slides with 90% glycerol and 20 mM Tris-HCl, pH 8.0. Images were taken at room temperature with a restoration microscope (DeltaVision RT; Applied Precision) using a 100x 1.40 NA Plan Apo objective and a filter set (Sedat Quad; Chroma Technology Corp.). Images were captured with a charge-coupled device camera (CoolSNAP HQ; Photometrics) with a z-optical spacing of 0.2 μm. Raw images were then deconvolved with the SoftWorx software (Applied Precision), and these were then processed, and PhotoShop (Adobe) was used to analyse the images.

### Time lapse

Cells were plated at 8 × 10^4^ cells/mL in a 24 well plate (Corning) at a volume of 500 μL. 16 hours later DMSO (control) or Cenp-E inhibitor (50 nM) were added. Cells were then imaged every 2 minutes using a Zeiss Axiovert 200 microscope, with an automated PZ-2000 stage (Applied Biosystems), with cells maintained at 37°C and a continuous flow of 5% CO_2_. Images were acquired with a 60x objective. All the shutters, filter wheels and point visiting were driven by MetaMorph software (Universal imaging). Images were taken with a camera (CoolSNAP HQ; Photometrics) and processed with Photoshop (Adobe), and Quicktime (Apple).

### FACS

The method was as previous [48]. After harvesting, samples were fixed in 100% ethanol at −20°C overnight, and after washing with PBS, cells were resuspended in propidium iodide (40 μg/mL) and RNase (50 μg/mL), leaving at room temperature for 30 minutes. Flow cytometric analysis was performed measuring DNA content of at least 10, 000 cells using a Cyan ADP (Beckman Coulter), and Summit 4.3 was used for data analysis.

### Proliferation and apoptosis assays

For the cell proliferation, caspase 3/7 activation and the phase imaging, cells were plated in a 96 well plate (Greiner Bio-One) at a density of 1 × 10^5^ cells/mL, with 100 μL per well. The IncuCyte ZOOM (Essen BioSciences) was used to image the cells, according to the manufacturer's instructions. Prism (GraphPad) was used for the analysis and fate profiling.

### Interphase FISH

HCT-116 cells were plated at a density of 8 × 10^4^ cells/mL onto glass coverslips. After drug treatment, cells were washed and 75 mM potassium chloride was added. Samples were fixed with methanol-acetic acid (3:1). Alpha-satellite probes for chromosomes 6 and 7 (MP Biomedicals and Cytocell) were used consistent with the manufacturers protocol. Chromosome signals in 300 nuclei were scored according to [16]. FISH images were acquired as 0.25 μm optical sections with the 60x 1.4 NA objective and are projections of four to five merged planes in the z-axis.

## SUPPLEMENTARY VIDEOS


