# Supplementary figures and images for: Cenp-E inhibitor GSK923295: Novel synthetic route and use as a tool to generate aneuploidy
Source: Oncotarget. 2015 Aug 6;6(25):20921–32. doi: 10.18632/oncotarget.4879 (PMC4673239; doi:10.18632/oncotarget.4879)

## SUPPLEMENTARY VIDEOS

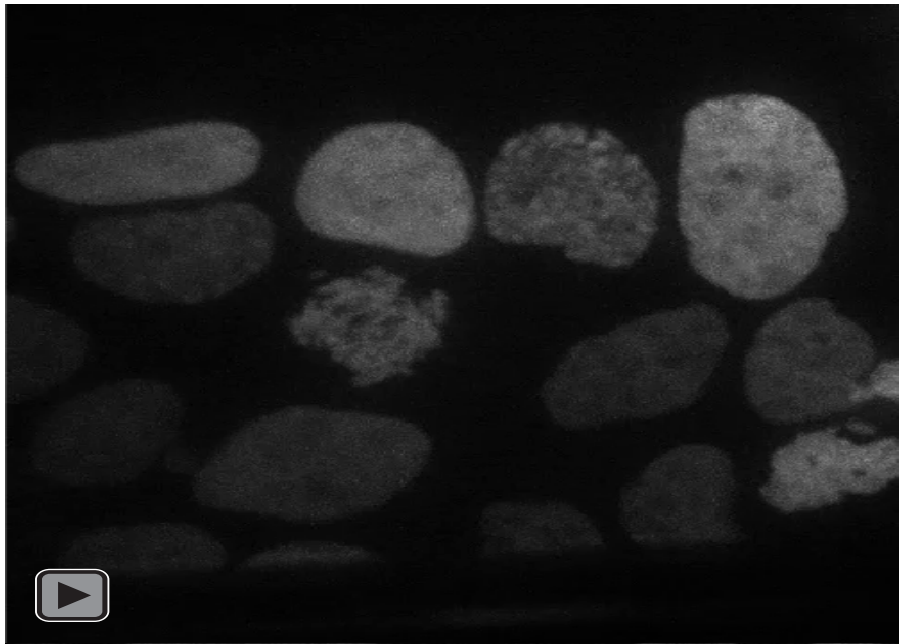

**Supplementary Movie S1:**

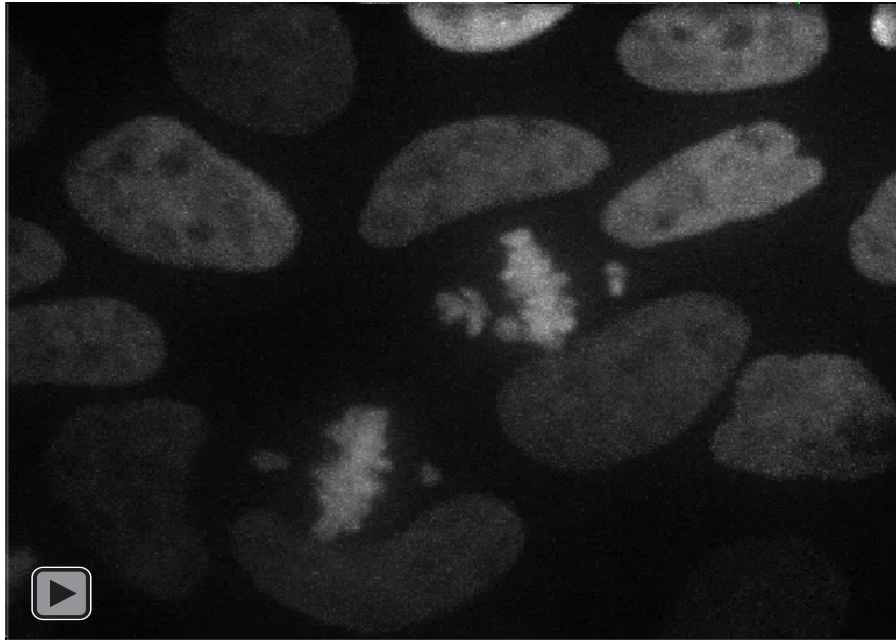

**Supplementary Movie S2:**

Supplement: Supplementary file 1 [file oncotarget-06-20921-s001.pdf]
